# The Effect of 40-Hz Light Therapy on Amyloid Load in Patients with Prodromal and Clinical Alzheimer's Disease

**DOI:** 10.1155/2018/6852303

**Published:** 2018-07-30

**Authors:** Rola Ismail, Allan K. Hansen, Peter Parbo, Hans Brændgaard, Hanne Gottrup, David J. Brooks, Per Borghammer

**Affiliations:** ^1^Department of Nuclear Medicine and PET Centre, Aarhus University Hospital, Aarhus, Denmark; ^2^Department of Neurology, Aarhus University Hospital, Aarhus, Denmark; ^3^Division of Neuroscience, Newcastle University, Newcastle, UK

## Abstract

Alzheimer's disease (AD) is a progressive neurodegenerative disorder. AD pathology is characterized by abnormal aggregation of the proteins amyloid-*β* (A*β*) and hyperphosphorylated tau. No effective disease modifying therapies are currently available. A short-duration intervention with 40 Hz light flicker has been shown to reduce brain A*β* load in transgenic mice. We aimed to test the effect of a similar short-duration 40 Hz light flicker regime in human AD patients. We utilized a Light Emitting Diode (LED) light bulb with a 40 Hz flicker. Six A*β* positive patients received 10 days of light therapy, had 2 hours of daily exposure, and underwent a postintervention PiB PET on day 11. After 10 days of light therapy, no significant decrease of PiB SUVR values was detected in any volumes of interest tested (primary visual cortex, visual association cortex, lateral parietal cortex, precuneus, and posterior cingulate) or in the total motor cortex, and longer treatments may be necessary to induce amyloid removal in humans.

## 1. Introduction

Alzheimer's disease (AD) pathology is characterized by abnormal aggregation of the proteins amyloid-*β* (A*β*) and hyperphosphorylated tau accompanied by brain inflammation in the form of microglial activation [1, 2]. No effective disease-modifying therapies are currently available for AD. To date, immunotherapy trials, with both passive antiamyloid and antitau antibodies and vaccines, while proving effective in lowering the abnormally aggregated protein load, have generally proved ineffective in improving cognitive status with one possible exception (*aducanumab*) though further trials are currently running in mild cognitive impairment and amyloid positive elderly subjects without cognitive symptoms [3, 4].

A recent animal study demonstrated a 50% decline in insoluble A*β* load in the cortex of an AD transgenic mouse model after a 1-hour exposure to 40 Hz noninvasive light stimulation each day for 7 days [5]. This is the first evidence that the induction of 40 Hz gamma frequencies enhances clearance of pathological protein inclusion in an AD model though, interestingly, higher and lower frequencies proved ineffective. Positron emission tomography (PET) radiotracers are now available to image* in vivo* aggregated A*β*, including ^11^C-Pittsburgh compound B (PiB) [6].

It is interesting and breaking knowledge if the results from the mouse studies can be replicated in humans as the theory behind AD is based on A*β* and microglial activation [7, 8]. Iaccaroni et al. showed that microglia were activated by light therapy reduced the A*β* load.

We have performed a pilot study with the specific hypothesis that daily exposure to short-duration 40 Hz light flicker stimulation would diminish the cortical A*β* load in AD and MCI subjects with abnormal PiB PET scans and, if it does, further studies are needed to explain the mechanisms behind.

## 2. Methods

### 2.1. Subjects

Five mild-to-moderate AD patients and one mild cognitive impairment (MCI) patient were recruited to the study. All subjects received their diagnoses from Dementia Clinics in Denmark in accordance with ICD-10 AD and Petersen MCI criteria [9]. All subjects were in treatment with donepezil.

Further inclusion criteria were as follows: (1) age: 50-85 years; (2) ≥7 years of education or working history. Exclusion criteria were as follows: (1) past history of concussive head injury or stroke; (2) previous history of epilepsy or current epilepsy; (3) current migraine; (4) contraindications to MRI; (5) significant systemic or psychiatric diseases; (6) other neurodegenerative disease; (7) use of medications/treatments which could influence neuropsychological testing, including electroconvulsive therapy; (8) participation in other clinical trials within 30 days of study entry.

### 2.2. Light Stimulus

We utilized a Light Emitting Diode (LED) 40 Hz light bulb (12.5 ms light on, 12.5 ms light off). The sharp on-off square-wave frequency flickering pattern was verified with an oscilloscope (Supplementary Figure 1A). Light flicker stimulation was tested one meter from a healthy 42-year-old male volunteer fitted with an EEG electrode montage (*SOMNOMedics, Randersacker, Germany*). A photic driving response was confirmed, i.e., presence of 40 Hz oscillations in frontal and occipital leads (Supplementary Figure 1B). Light stimulation was performed with the patients seated in their own home in the dark. The background illumination was measured to be 0.5-0.6 LUX at the locations where patients received light stimulation. Thus, maximal light-dark contrast was ensured for the light flicker stimulation. The light bulbs' illumination was measured before and after light therapy using a ISO-TECH ILM 1335 (*ISO-TECH, Midrand, South Africa*) and was found to be stable with no significant loss of efficiency, and mean intensity measurements of the light sources were pretreatment Lux =1815 ± 94.6 and posttreatment Lux= 1737 ± 68.3.

Caregivers were instructed to ensure that patients received a continuous 60-minute 40 Hz light stimulation twice daily (1 hour morning, 1 hour evening) for 10 consecutive days. No other light sources were allowed during treatment (computer, tablet, mobile phone, television, etc.) but the patients were allowed to read or do other manual chores at a distance of 0.5-1 meter from the LED light source. The patients were also asked to look directly into the light source for a minimum of 5 minutes during every session. Patients and caregivers were provided with a stopwatch and instructed to record the total time duration of each light session and the time spent looking directly at the light source. Postintervention PiB scans were for all subjects performed the day following the final intervention day.

### 2.3. Imaging

Isometric 1 mm T_1_-weighted magnetic resonance imaging (MRI) was performed with a Skyra 3T system (*Siemens, Erlangen, Germany*).

^11^C-PiB PET was performed with a High Resolution Research Tomograph (*ECAT HRRT, CTI/Siemens, Knoxville, TN*) according to a previously published scan protocol [1]. The pretreatment scans were performed at different time before treatment start, while the posttreatment scans all were performed on the day following the end of the light-therapy, i.e., on day 11. A mean dose of 407 ± 13.8 MBq (for poststimulation scans) and 424 ± 15.3 MBq (for the prestimulation scans) ^11^C-PiB was injected, and list-mode PET was acquired for 40-90 minutes postinjection. Data was subsequently rebinned into five frames of 10 min each.

### 2.4. Image Analysis

The PNEURO toolbox from PMOD version 3.6 (*PMOD, Zürich, Switzerland*) was used for image processing and analysis. MRI volumes were segmented into grey matter (GM), white matter (WM), and cerebrospinal fluid (CSF). GM masks were convolved with a probabilistic atlas [10] to individualize VOIs to individual GM. Volumes defining primary visual cortex and precuneus were manually added to the atlas. Mean 50-90 min PiB images were coregistered to individual anatomical MRI, and the transformation matrices from the individual's native MRI space to MNI space were applied to the PET images. PiB SUVR images were generated by dividing each voxel value in the averaged PiB images by the mean PiB uptake in the individual's cerebellar GM VOI [11]. PiB SUVR values were sampled from 5 VOIs: primary visual cortex, visual association cortex, lateral parietal, precuneus, and posterior cingulate cortices. To minimize spill-in/spill-out, no smoothing was applied prior to extraction of PiB SUVR values from the VOIs. Correct placement of each VOI in each subject was visually confirmed on both MRI and PET in atlas space.

### 2.5. Statistical Analysis

Data is presented as mean ± standard deviation (SD). Data were analysed using STATA version 14.2 (StataCorp LP, Texas, USA). Paired t-tests were used to test pre-/postchanges in PiB SUVRs with a* p*<0.05 threshold for statistical significance.

We assumed a maximal test-retest coefficient-of-variance of cortical PiB uptake of 12 % [12, 13]. A power calculation with alpha=0.05 showed that we would be able to detect a 20% decline in PIB retention with 90% power.

## 3. Results

Demographic, clinical, and individual regional SUVR data are presented in Table 1. Median duration from the baseline PET scan to the poststimulation scan was 89 days (range: 18-283), while all poststimulation scans were performed one day after end stimulation. The average time duration of the light therapy sessions was 61.5 ± 4.8 minutes per session, during which the patients looked directly at the light source for 39.3 ± 21.5 minutes per session (range: 10-65 minutes).

**Figure 1:**
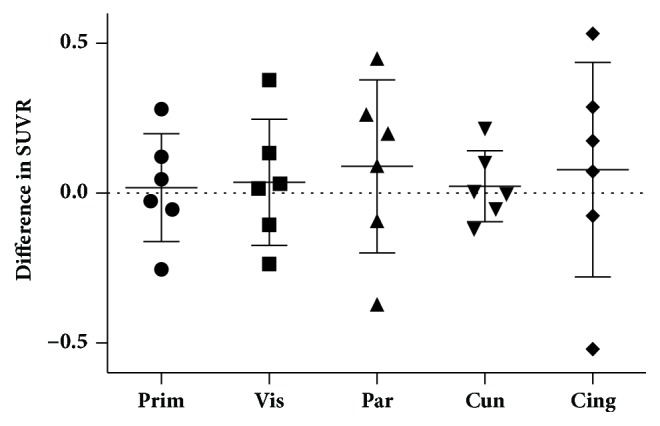
Mean PiB SUVR differences in five cortical regions. No significant difference in SUVR before and after light stimulation was seen (Prim = primary visual, Vis = visual association, Par = Parietal lobe, Cun = Cuneus, and Cing = posterior cingulate).

No significant mean PiB SUVR differences were detected in any of the five VOIs on comparing pre- and post-stimulation PET (*p* > 0.48; Figure 1). Additionally, no difference was seen in total cortical ^11^C-PiB uptake using a total cortical GM VOI (pre-SUVR 2.314 ± 0.44; post-SUVR 2.378 ± 0.41;* p* = 0.7).

**Figure 2:**
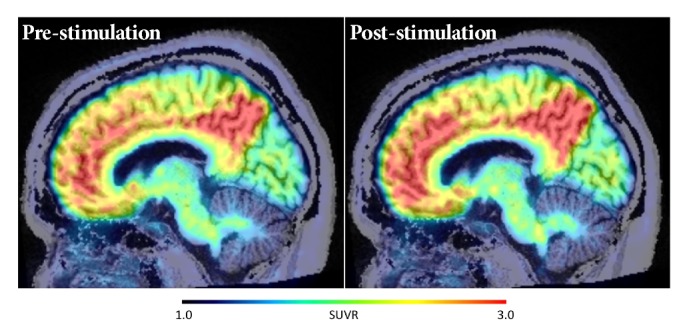
Average ^11^C-PiB SUVR images of the six subjects in the pre- and poststimulation conditions. No significant differences were seen anywhere in cortical regions.

Figure 2 displays averaged pre- and poststimulation ^11^C-PiB SUVR images of the six subjects. Concurrent negative findings were found using the pons and WM as reference regions (data not shown).

## 4. Discussion

This pilot study examined whether the marked 50% reduction of cortical A*β* seen in transgenic AD mice after short-duration exposure to 40 Hz light stimulation would translate into human AD patients. However, we saw no effect of 40 Hz light-therapy on A*β* load in the primary visual cortex or any other region of early AD patients.

A test-retest study showed a mean difference in PiB-uptake of 3-7 % in cortical regions within 20 days [13]. The greatest difference of 12.7 % was seen in the striatum [13]. We based our power calculation on a 12 % test-retest variance of PiB PET scans [12, 13], yielding 90% power to detect a 20% decline in SUVR. We believe the 20% estimate was conservative, as another PiB PET study reported lower regional test-retest difference within 20 days. Thus, if there was an effect of 40 Hz light stimulation on A*β* load it was most likely less than 20% and not comparable to the 50% reduction seen in AD mice.

It is possible that a longer-duration light therapy regime could have had a measurable effect. It is also possible that a therapeutic effect in humans can only be achieved by looking directly into the light source and not only by being exposed to a flashing environment, although the published mouse study applied only the latter. A recent PiB PET study showed that injections of the human monoclonal antibody* aducanumab*, which targets aggregated A*β*, significantly reduced cortical PiB signal over 52 weeks [4]. At 26 weeks only 60-70% of the full effect had been achieved. It is questionable whether the effect of aducanumab would have been detectable after only 10 days of therapy. The same may hold true for 40 Hz light stimulation.

The study had several limitations: the duration of light stimulation was brief, but this limitation was by design. Two subjects had a significant gap between the pre- and postintervention scans (>3 months), which may have blunted statistical power. Nevertheless, it can be concluded that light-therapy induced reductions in cortical amyloid load of the magnitude seen in mouse models (>50%) do not occur in human patients.

The gamma entrainment, in form of the EEG response to the 40 Hz light flicker, was tested on a young healthy subject but not tested on our elderly subjects. Future studies should preferably test the gamma entrainment on each individual test subject.

All our subjects, including the MCI subject, were treated with acetylcholine esterase inhibitors (donepezil); we cannot exclude an effect of donepezil on gamma entrainment, although it seems unlikely that a treatment, which restores cognitive functions, would have a negative effect on the brains neurophysiological condition.

Our findings also highlight the difference between transgenic rodent models of AD and the human condition. Although mice models are a promising tool for investigating efficacy of anti-A*β* interventions, there are anatomical and physiological differences between humans and rodents and the interspecies differences in A*β* aggregation and toxicity might explain the difference in efficacy of therapeutic interventions between rodents and human [14].

In summary, a short-duration 40 Hz light therapy regime did not decrease cortical amyloid load in patients with early Alzheimer's disease. Future studies are needed to investigate whether extended duration therapies could have beneficial effects.

## Figures and Tables

**Table 1:** Demographic, clinical, and PET data.

**Subject no.**	**1**	**2**	**3**	**4**	**5**	**6**	**Median [range]**
Age (years)	70	67	73	54	85	80	71.5 [54-85]

Gender (F/M)	F	M	M	M	M	M	(1/5)

CDR	1	0.5	0.5	0.5	0.5	0.5	0.5 [0.5; 1]

SOB	5.5	3.5	2	2.5	1.5	2.5	2.5 [1.5; 5.5]

MMSE	19	25	25	27	26	27	25.5 [19; 27]

AD/MCI	AD	AD	MCI	AD	AD	AD	(5/1)

Disease dur. (months)	10	3		9	20	13	10 [3; 20]

Pre-post scan duration (month)	9.5	1.1	0.6	1.1	13.8	4.0	2.5 [0.6; 13.8]

**PiB SUVR:**							**Mean [95 **%** CI]**

Visual pre	3.13	2.12	1.27	2.39	1.67	1.18	1.96 [1.18; 2.74]
Visual post	3.41	2.10	1.39	2.14	1.61	1.22	1.98 [1.15; 2.81]
Difference (%)	9.0 %	-1.3 %	9.5 %	-10.6 %	-3.2 %	4.0 %	1.2 % [-7.0; 9.4]

Vis. Ass pre	2.83	2.10	1.45	2.07	1.89	1.34	1.94 [1.39; 2.51]
Vis. Ass post	3.21	1.99	1.58	1.84	1.92	1.35	1.98 [1.30; 2.66]
Difference (%)	13.4 %	-5.0 %	9.2 %	-11.4 %	1.7 %	1.1 %	1.5 % [-8.0; 11.0]

Parietal pre	2.73	2.80	2.38	2.94	2.54	1.74	2.49 [2.03; 2.95]
Parietal post	2.99	2.71	2.83	2.57	2.34	1.83	2.58 [2.16; 3.0]
Difference (%)	9.6 %	-3.3 %	18.9 %	-12.6 %	-7.9 %	5.2 %	4.4 % [-7.1; 15.9]

Precuneus pre	3.31	3.19	2.26	3.44	2.59	2.10	2.81 [2.21; 3.41]
Precuneus post	3.58	2.95	2.72	2.96	2.83	2.19	2.87 [2.40; 3.34]
Difference (%)	8.1 %	-7.5 %	20.0 %	-13.8 %	9.1 %	4.4 %	3.4 % [-9.3; 16.2]

Post. Cing. Pre	3.25	3.26	2.41	3.61	2.56	1.95	2.84 [2.18; 3.50]
Post. Cing. Post	3.54	3.19	2.94	3.09	2.63	2.12	2.92 [2.40; 3.34]
Difference (%)	8.8 %	-2.3 %	22.1 %	-14.4 %	2.8 %	9.0 %	4.3 % [-8.6; 17.2]

Global pre	2.87	2.57	2.00	2.69	2.19	1.61	
Global post	3.17	2.46	2.33	2.35	2.30	1.67	
Difference (%)	10.6 %	-4.1 %	16.5 %	-12.5	5.4 %	4.1 %	

CDR: Clinical Dementia Rating; SOB: CDR sum-of-boxes; MMSE: minimental state examination; AD: Alzheimer's disease; MCI, mild cognitive impairment; SUVR: standardized uptake value ratio; pre: before light stimulation; post: after light stimulation.

## Data Availability

The authors confirm that the data supporting the findings of this study are available within the article and its supplementary materials.

## Supplementary Materials

Supplementary Materials Supplementary Figure 1: A. A 40 Hz sharp-wave pattern was confirmed for all utilized light bulbs using an external light sensor. B. The EEG response in an occipital and frontal EEG lead elicited by 40 Hz light flicker stimulation in a 42-year-old healthy subject. Red arrows indicate 40 Hz photic driving.
