# Supplementary material for: The Effect of 40-Hz Light Therapy on Amyloid Load in Patients with Prodromal and Clinical Alzheimer's Disease
Source: Int J Alzheimers Dis. 2018 Jul 30;2018:6852303. doi: 10.1155/2018/6852303 (PMC6091362; doi:10.1155/2018/6852303)
Supplement: Supplementary Materials — Supplementary Figure 1: A. A 40 Hz sharp-wave pattern was confirmed for all utilized light bulbs using an external light sensor. B. The EEG response in an occipital and frontal EEG lead elicited by 40 Hz light flicker stimulation in a 42-year-old healthy subject. Red arrows indicate 40 Hz photic driving. [file 6852303.f1.docx]

**Supplementary Materials**


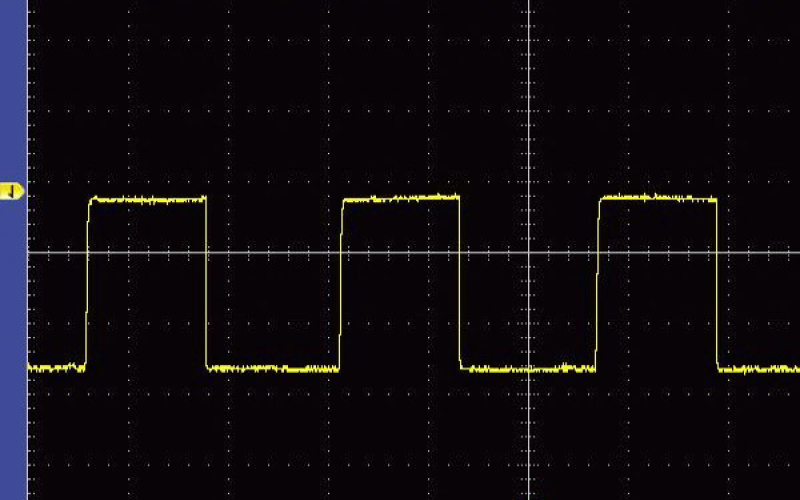

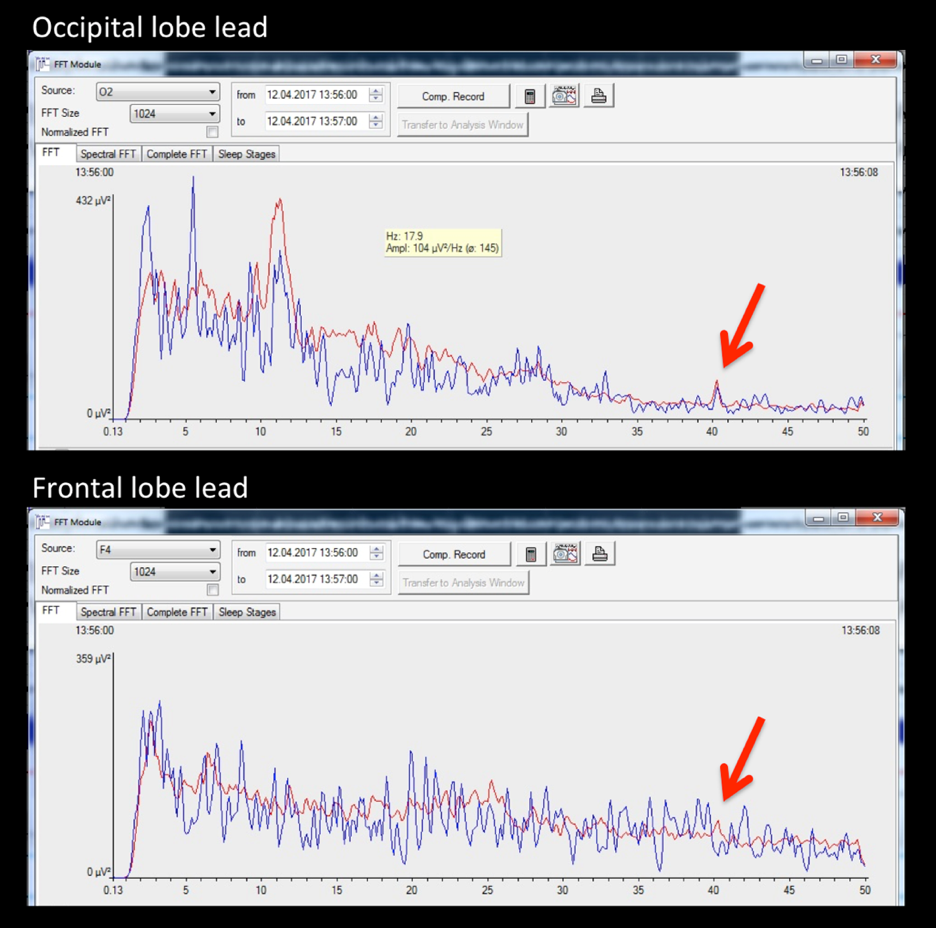


**A**

**B**

**A**

**Supplementary Figure 1:**

**A.** A 40 Hz sharp-wave pattern was confirmed for all utilized light bulbs using an external light sensor.

**B.** The EEG response in an occipital and frontal EEG lead elicited by 40 Hz light flicker stimulation in a 42-year old healthy subject. Red arrows indicate 40 Hz photic driving
